# Microwave‐Assisted Selective Hydrogenation of Furfural to Furfuryl Alcohol Employing a Green and Noble Metal‐Free Copper Catalyst

**DOI:** 10.1002/cssc.201601398

**Published:** 2016-12-16

**Authors:** Pedro N. Romano, João M. A. R. de Almeida, Yuri Carvalho, Peter Priecel, Eduardo Falabella Sousa‐Aguiar, Jose A. Lopez‐Sanchez

**Affiliations:** ^1^ Technology of Chemical and Biochemical ProcessesFederal University of Rio de JaneiroAv. Horácio Macedo 203021941-909Rio de JaneiroBrazil; ^2^ Department of ChemistryUniversity of LiverpoolCrown StreetL69 7ZDLiverpoolUnited Kingdom

**Keywords:** copper, furfural, furfuryl alcohol, hydrogenation, microwaves

## Abstract

Green, inexpensive, and robust copper‐based heterogeneous catalysts achieve 100 % conversion and 99 % selectivity in the conversion of furfural to furfuryl alcohol when using cyclopentyl‐methyl ether as green solvent and microwave reactors at low H_2_ pressures and mild temperatures. The utilization of pressurized microwave reactors produces a 3–4 fold increase in conversion and an unexpected enhancement in selectivity as compared to the reaction carried out at the same conditions using conventional autoclave reactors. The enhancement in catalytic rate produced by microwave irradiation is temperature dependent. This work highlights that using microwave irradiation in the catalytic hydrogenation of biomass‐derived compounds is a very strong tool for biomass upgrade that offers immense potential in a large number of transformations where it could be a determining factor for commercial exploitation.

Furfural (FAL) has recently gained a lot of attention as a key platform chemical derived from hemicellulosic biomass.^1^, ^2^ FAL hydrogenation may lead to several value‐added products such as furfuryl alcohol (FOL), tetrahydrofurfuryl alcohol (THFA), 2‐methylfuran (MF), and 2‐methyltetrahydrofuran (MTHF) as depicted in Scheme 1.^3^, ^4^, ^5^ The development of an adequate catalytic system to control the selectivity to a desired product is a critical step that still deserves as much attention as developing greener and highly active catalysts.

**Scheme 1:**
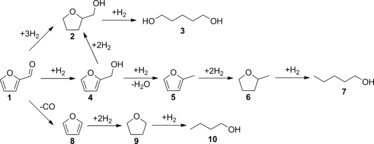
Main products obtained from FAL hydrogenation and side reactions: FAL (**1**), THFA (**2**), 1,5‐pentanediol (**3**), FOL (**4**), MF (**5**), MTHF (**6**), 1‐pentanol (**7**), furan (**8**), tetrahydrofuran (**9**), and 1‐butanol (**10**).

FOL is an important furan derivative that has application in the production of resins;^6^ as an intermediate for the production of lysine, ascorbic acid, and lubricants;^6^, ^7^ as well as a hypergolic fuel in rocketry.^8^ FOL is currently produced on industrial scale by liquid‐ or vapor‐phase hydrogenation of FAL employing a copper chromite catalyst, with an annual production of 400 000 t.^9^ The main drawbacks of the current process are: the toxicity of the catalyst used; relatively high pressures of H_2_, in case of liquid‐phase hydrogenation; and high temperatures, meaning high energy consumption for the vapor‐phase hydrogenation.^9^

The majority of the scientific papers addressing the production of FOL either use harsh conditions;^10^, ^11^, ^12^, ^13^, ^14^, ^15^, ^16^, ^17^ high pressures of H_2_;^14^, ^18^ or noble metals,^19^, ^20^, ^21^ which are becoming scarce, more expensive, and raise many sustainability concerns. Kyriakou and co‐workers.^7^ recently tested Pt nanoparticles supported on γ‐Al_2_O_3_, SiO_2_, CeO_2_, and ZnO, obtaining 80 % conversion of FAL and 99 % selectivity to FOL after 7 h at 50 °C using methanol as solvent and Pt/γ‐Al_2_O_3_ as catalyst. On the other hand, Jérôme and co‐workers^9^ presented a very promising result using a partially recyclable Co/SBA‐15 catalyst that achieves 88 % yield of FOL after 1.5 h at 150 °C and 20 bar of H_2_. However, the conversion decreased from 92 to 81 % between the first two cycles and continued to decrease slowly in subsequent cycles. Xie and co‐workers.^14^ reported a maximum yield of FOL of 90 % after 5 h at 160 °C and 90 bar of H_2_ using a Cu–Fe catalyst, which was also active for the hydrogenation of levulinic acid.

Cyclopentyl methyl ether (CPME) emerged in the past few years as a new, safe, and green solvent alternative to other ether solvents such as 1,4‐dioxane, tetrahydrofuran, and MTHF. This is because CPME offers low peroxide formation and toxicity, narrow explosion range, high hydrophobicity and stability under acidic and basic conditions, and a relatively high boiling point.^22^, ^23^, ^24^, ^25^, ^26^, ^27^ Overall, CPME addresses eight out of the twelve principles of Green Chemistry.^28^ Therefore, herein, CPME was evaluated as a potential green solvent for FOL production yet unexplored for this reaction.

Recently, microwave‐assisted reactions have become more popular among the scientific community. Microwave irradiation enables a fast, uniform, and efficient dielectric heating of the reaction media, generating an increase in reaction rates as well as reducing the energy consumption.^29^, ^30^, ^31^, ^32^, ^33^, ^34^ The use of this technique represents a breakthrough in terms of sustainability, efficiency, development of new materials, and cost reduction.^31^, ^35^, ^36^, ^37^ To the best of our knowledge, the use of microwave irradiation has not been reported in FAL hydrogenation aiming at producing FOL.

Herein, we report a green and efficient catalytic system for the selective hydrogenation of FAL to FOL that combines a more sustainable, highly active, and recyclable catalyst, Cu/TiO_2_, with using a green solvent (CPME) under conventional and microwave heating under H_2_ pressure.

Powder X‐ray diffraction (PXRD) patterns of the calcined and reduced Cu/TiO_2_ catalysts are shown in Figure 1. In the case of the reduced catalyst, we identified only diffraction peaks related to the cubic Cu^0^ phase, that is, 2*θ*=43.1° (111), 50.2° (200), and 73.9° (220) (JCPDS 00‐004‐0836). Concerning the calcined sample, only characteristic peaks of CuO, 2*θ*=35.3° (002) and 38.5° (111) were observed, as expected (JCPDS 00‐048‐1548). The remaining diffraction peaks could be attributed to the anatase (JCPDS 00‐021‐1272) and rutile (JCPDS 01‐075‐1748) phases of TiO_2_.

**Figure 1:**
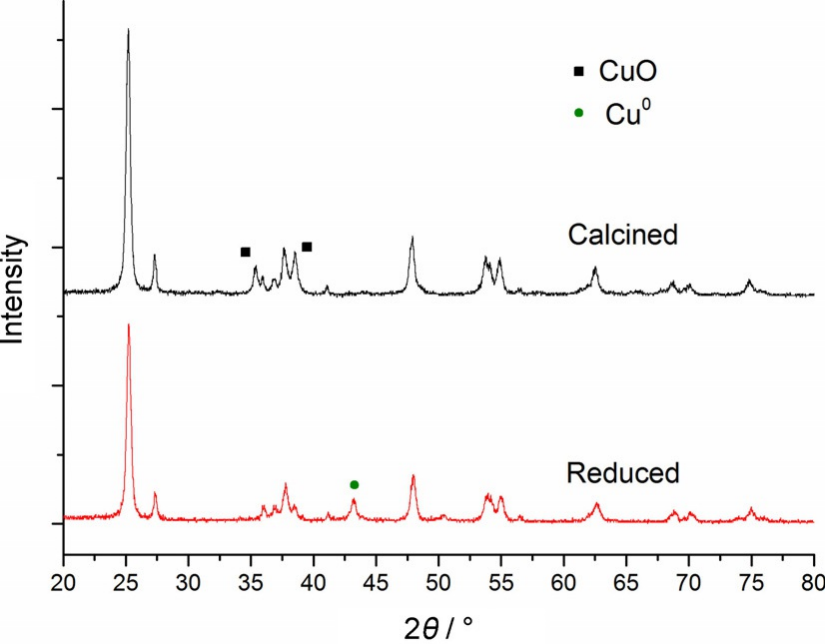
Powder X‐ray diffraction of the calcined and reduced Cu/TiO_2_ catalyst.

Thermal gravimetric analysis (TGA) was performed to assess the evolution of the decomposition of the copper precursor, as depicted in Figure S1 in the Supporting Information. The results show that the calcination conditions employed (400 °C, 4 h) were enough to decompose all of the copper precursor salt.

To evaluate the reducibility of the calcined catalyst, temperature‐programmed reduction (TPR) analysis with hydrogen was performed (Figure 2). In the case of Cu/TiO_2_, two sharp and narrow peaks were observed at 127 and 175 °C. It is common to assign the first reduction peak to the stepwise reduction of Cu^2+^ to Cu^1+^ whereas the second peak should correspond to the reduction of Cu^1+^ to Cu^0^.^38^, ^39^, ^40^ However, another possible explanation would be based on the existence of different copper species present on the support. Therefore, the first peak could be owed to the reduction of highly dispersed copper nanoparticles whereas the second could correspond to the reduction of bulk copper‐oxide species or larger particles.^41^, ^42^ TEM of the freshly reduced Cu/TiO_2_ catalyst along with particle‐size distribution are shown in Figure 3. It is possible to see that the catalyst is comprised of relatively small copper nanoparticles with a narrow size distribution, having an average size of approximately 2.2 nm. Figure S2 shows a TEM image of the TiO_2_ support, highlighting the lattice *d*‐spacing corresponding to the (101) plane of the anatase phase.

**Figure 2:**
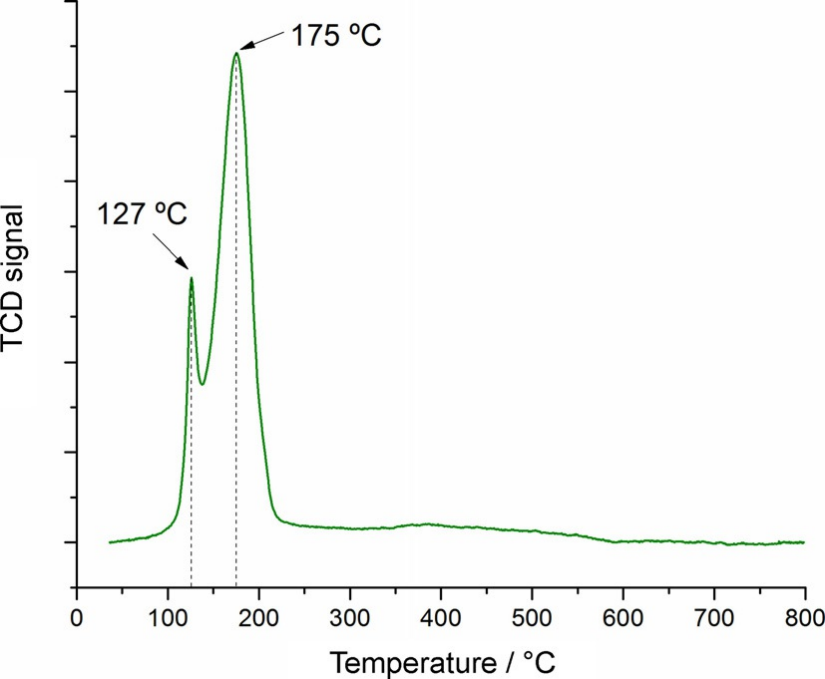
H_2_‐TPR curve of the calcined Cu/TiO_2_ catalyst. Experimental conditions: 5 % H_2_ in N_2_, 50 mL min^−1^, 10 °C min^−1^, 50 mg sample.

**Figure 3:**
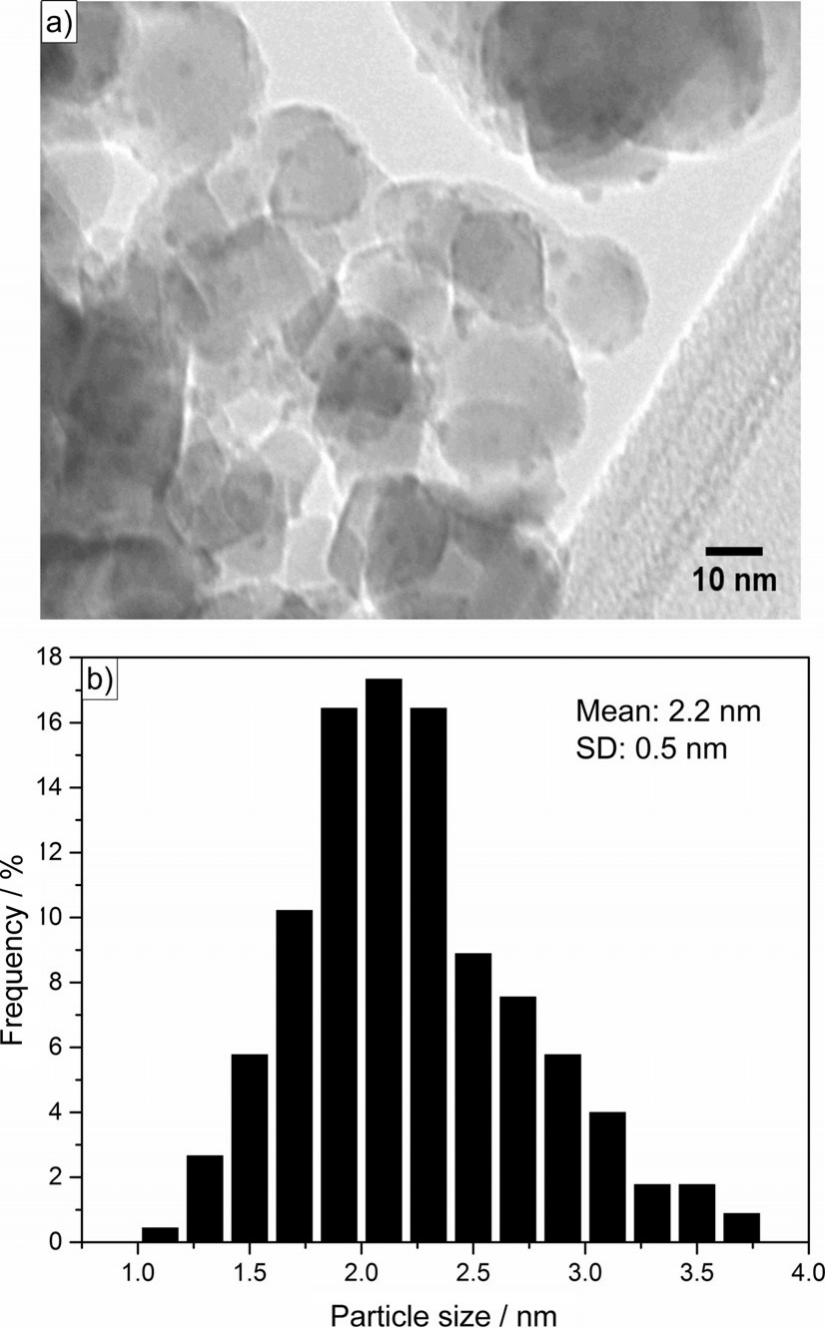
a) TEM micrograph of freshly reduced Cu/TiO_2_ catalyst. b) Particle‐size distribution for Cu/TiO_2_ as catalyst. SD stands for standard deviation.

Catalytic tests were performed using the synthesized Cu/TiO_2_ catalyst in a microwave reactor at 125 °C and 10 bar of H_2_, as depicted in Table 1. The blank test done in the absence of catalyst showed a negligible FAL conversion at these conditions. We can see that the catalyst is highly active and highly selective towards FOL, reaching 97 % conversion with 100 % selectivity to FOL in 2 h. Moreover, 99 % yield of FOL was obtained after 3 h with a minor formation of MF due to FOL hydrogenolysis. These are particularly good results considering that low hydrogen pressures and low temperatures were utilized.

**Table 1:** FAL selective hydrogenation to FOL.^[a,b]^

Entry	Reaction time [min]	FAL Conversion [%]^[c]^	FOL Selectivity [%]^[c]^	MF Selectivity [%]^[c]^	FOL Yield [%]	MF Yield [%]
1	20	3	100	0	3	0
2	60	46	100	0	46	0
3	120	97	100	0	97	0
4	180	100	99	1	99	1
5	240	100	99	1	99	1

[a] Experimental conditions: reactions performed in the microwave reactor at 125 °C, 10 bar of H_2_, 10 mg of 10 % Cu/TiO_2_, 5 mL of a 40 mm FAL solution in CPME. [b] Reaction without catalyst at the same conditions resulted in a negligible FAL conversion (ca. 1 %). [c] Determined by GC.

It is well known that deactivation of copper‐based catalysts is one of the major drawbacks for copper catalysis and it is very often neglected; thus after these promising preliminary results, we decided to test the recyclability of the Cu/TiO_2_ catalyst. Figure 4 shows the results of the three cycles of recycling tests performed with the Cu/TiO_2_ catalyst.

**Figure 4:**
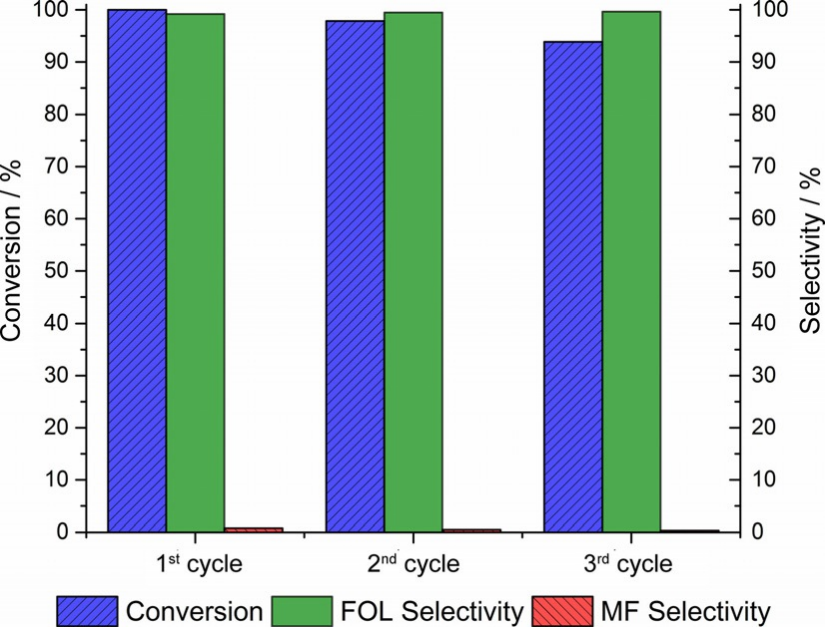
Recycling tests for FAL hydrogenation to FOL over Cu/TiO_2_. Experimental conditions: 125 °C; 10 bar of H_2_; 180 min; 10 mg of 10 % Cu/TiO_2_; 5 mL of a 40 mm FAL solution in CPME; microwave reactor.

We can observe that the catalyst is reusable under the tested conditions, showing a minor decrease in conversion from 100 to 94 % after the third reaction cycle, maintaining, however, a high selectivity to FOL. The most common reason for the deactivation of copper‐based catalysts is metal nanoparticle sintering, which reduces the metallic surface area available for catalysis. Concerning the Cu/TiO_2_ catalyst, no signs of severe sintering were observed, which we believe is owed to a strong metal–support interaction (SMSI),^41^, ^42^ or an electronic metal–support interaction (EMSI), as proposed by Lykhach et al.^43^ and Campbell.^44^ This metal–support interaction would allow the catalyst to retain the majority of its activity, preventing particle sintering. Similar effects were reported in the literature that support our conclusions for the hydrogenation of FAL under microwave heating in the presence of the Cu/TiO_2_ catalyst.^42^, ^45^, ^46^ In this sense, we attribute the slight decrease in activity between each cycle to a very small sintering of the Cu nanoparticles or to a minor surface oxidation occurring during the recycling process. The combination of a high activity/ selectivity with good stability makes this material a promising catalyst for FOL synthesis from FAL.

Subsequently, we decided to perform an additional set of reactions under conventional heating in an autoclave at the same conditions employed in the microwave studies: 125 °C and 10 bar of H_2_. The comparison between the reaction profile using both heating systems is shown in Figure 5.

**Figure 5:**
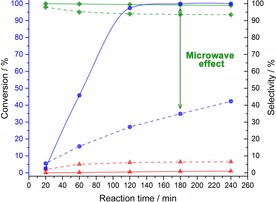
Comparison between FAL hydrogenation over Cu/TiO_2_ under conventional heating (dashed lines) and microwave irradiation (solid lines). Legend: conversion (•), FOL selectivity (⧫) and MF selectivity (▴). Parr reactor conditions: 125 °C; 10 bar of H_2_; 60 mg of 10 % Cu/TiO_2_; 30 mL of a 40 mm FAL solution in CPME. Microwave reactor conditions: 125 °C; 10 bar of H_2_; 10 mg of 10 % Cu/TiO_2_; 5 mL of a 40 mm FAL solution in CPME.

The differences between the two systems are striking: the reaction carried out under microwave heating has a much higher reaction rate, reaching full conversion at 180 min; in contrast, only 35 % conversion is achieved under conventional heating. Although microwave reactors have been used for a few years in organic reactions, the real influence and effects that microwave irradiation has upon the reaction medium are not yet completely understood and these microwave effects are particularly complex for reactions catalyzed by solid catalysts.^47^, ^48^ The existence of “non‐thermal” microwave effects or specific microwave effects is still a very controversial topic, and our findings do not show evidence toward a definitive conclusion.^48^, ^49^, ^50^ However, we believe that our results can be explained by means of thermal effects only.^51^ Microwaves have the ability, through dielectric heating, to selectively heat specific parts of the reaction medium, which may lead to higher reaction rates and changes in selectivity.^30^, ^32^, ^49^ Several authors observed the formation of hot spots in heterogeneous catalysts during microwave irradiation as a result of the materials’ high dielectric constants and loss tangent.^31^, ^32^, ^51^, ^52^ This could increase the temperature of the active sites of the catalysts locally above the temperature of the bulk, which could explain higher reaction rates.^31^, ^47^, ^51^ Following this hypothesis, TiO_2_ has a high dielectric constant of approximately 50, which suggests that it absorbs microwave irradiation very well, getting superheated above the solvent bulk temperature.^47^ However, we have not come to a conclusion as to how this effect could explain the enhancement in selectivity we observe in Figure 5 as hot spots would typically result in a loss of selectivity. Indeed, the reaction in the microwave is not only faster but also hinders the consecutive conversion of methyl furfural to methyl furan resulting in almost 100 % selectivity. We also know that higher reaction temperatures result in loss of selectivity at iso‐conversion (Figures S3 and S4); this would suggest that hot spots should have a negative effect on selectivity as well. Interestingly, Holzgrabe and co‐workers^52^ have proposed that when hydrogen is adsorbed at the catalyst surface, a dipole moment can be induced, which would allow the microwaves to interact with the adsorbed hydrogen. Although we have no evidence for such phenomenon taking place during reaction, we cannot exclude that it is responsible for the unexpected enhancement in selectivity we observe. In any case, the choice of support plays a crucial role, not only improving the catalyst stability, preventing nanoparticles sintering, but also boosting the reaction rate when coupled with microwave irradiation. For practical applications, and despite the very mild temperatures utilized, it is important to stress that the time required to reach the temperature set point in microwave‐assisted reactions was usually less than 3 min. In contrast, the reactions under conventional heating took 15–20 min to reach the same temperature.

Moreover, we decided to study the effect of the reaction temperature for both systems. Therefore, we carried out another set of reactions at 150 and 175 °C (Figures S3 and S4) and 10 bar of H_2_ under conventional and microwave heating. We see in Figure S3 that the disparities between reaction rates of the two systems at 150 °C are less pronounced compared to the reactions performed at 125 °C. Surprisingly, at 175 °C (Figure S4) no significant differences in conversion and selectivities were observed. This observation could also be attributed to thermal effects owed to the metal–support superheating. At low temperatures, the reactions proceed slowly because of energy limitations; therefore when microwave irradiation is employed, hot spots generate localized higher temperatures in the metal and support, enhancing the reaction rate. At higher temperatures, the microwave heating effect is attenuated as the available energy starts to be high enough to quickly convert the molecules, gradually decreasing the differences between both systems until no significant microwave‐irradiation effect is noticeable.

In conclusion, we have shown that a simple impregnation method provides a cheap, active, stable, and sustainable catalyst (Cu/TiO_2_), that works at low temperatures (125 °C) and low H_2_ pressures (10 bar of H_2_) for the furfural (FAL) hydrogenation to furfuryl alcohol (FOL). High yields of FOL were achieved (>99 %), employing cyclopentyl methyl ether (CPME) as a promising green solvent for hydrogenation reactions. In addition, we have assessed and compared the differences regarding the reaction evolution under conventional heating and microwave irradiation at different temperatures. We have observed much higher reaction rates under microwave irradiation at mild temperatures, which are temperature dependent and could be explained by localized thermal effects. We can conclude that the system composed of Cu/TiO_2_ as catalyst, CPME as solvent, and microwave irradiation as heating source is a promising, efficient, and green system for FOL production. Importantly, microwave dielectric heating has the potential to be a great tool, aiming at improving overall reaction conditions and ensuring high reaction rates for bio‐derived reactions. Furthermore, this work is a first example highlighting that using pressurized microwave reactors in the catalytic hydrogenation of biomass‐derived compounds can offer huge advantages in terms of conversion and selectivity and we believe this offers great potential in a large number of hydrogenations as well as oxidative transformations, where it could be determinant for commercial exploitation.

## Experimental Section

CPME (≥99.9 %) and Cu(NO_3_)_2_ puriss. pa. (99–104 %) were purchased from Sigma–Aldrich. TiO_2_ (Aeroxide^TM^ P25) was purchased from Fisher Scientific. All reagents were used as received.

The Cu/TiO_2_ catalyst was prepared using a simple wet‐impregnation method. The amount of dissolved Cu(NO_3_)_2_ precursor was calculated, aiming at achieving a 10 % copper loading. After the impregnation, the catalyst was dried overnight at 100 °C. The Cu/TiO_2_ catalyst was calcined at 400 °C for 4 h under air. After calcination and prior to all catalytic tests, the catalyst was reduced at 400 °C for 4 h under flow of pure H_2_.

PXRD patterns were recorded in transmission mode using a PANalytical X′Pert Pro HTS diffractometer with a slit of 0.04° from 2*θ*=4 to 90° using a CuK_α_ radiation. TPR analyses were performed using a Micromeritics AutoChem II 2920 equipped with a thermal conductivity detector. The samples (50 mg) were heated up to 800 °C at 10 °C min^−1^ at a flow of 20 mL min^−1^ of 5 % H_2_ in N_2_. TEM characterization was performed using a JEOL JEM 2100 LaB6 instrument at 200 kV accelerating voltage. The samples were sonicated in methanol and supported on holey carbon film on gold grids (300 mesh). Particle‐size distributions were determined by counting at least 200 particles.

The reactions performed under conventional heating were carried out in a Parr reactor Series 4590 with a volume of 50 mL. The reactor was loaded with a stock solution of FAL in CPME (40 mm, 30 mL) and the catalyst (60 mg). The vessel was then closed, purged five times with nitrogen and heated up to the desired temperature while stirring at 1000 rpm. Finally, after reaching the set temperature, the reactor was pressurized with 10 bar of H_2_ and the reaction was considered to have started.

A CEM Discover SP microwave reactor was employed to perform the microwave‐assisted hydrogenation reactions. The reactor was equipped with a gas addition kit containing an in situ fiber optic temperature control and a 10 mL reaction vessel operable up to 200 °C and 14 bar. The reactor was loaded with the same stock solution (5 mL) used in the Parr reactor and the catalyst (10 mg). Finally, the vessel was purged five times, loaded with the desired H_2_ pressure and heated up at the maximum stirring speed available. The reaction was considered to have started as soon as the temperature ramp started and the temperature set point was usually reached in less than 3 min.

The reaction products were identified using GC–MS (Agilent 5975, HP‐5 ms capillary column) and quantitatively determined using GC (Agilent 7820A) employing a flame ionization detector and a HP‐5 capillary column (30 m×0.32 mm×0.25 μm). The carrier gas used was N_2_ at a flow of 2 mL min^−1^.

Recycling tests were performed in the following manner: after each reaction, the catalyst was recovered by centrifugation, washed three times with methanol, dried, and then recharged in the reactor following the same procedure as described before.

## Supporting information

As a service to our authors and readers, this journal provides supporting information supplied by the authors. Such materials are peer reviewed and may be re‐organized for online delivery, but are not copy‐edited or typeset. Technical support issues arising from supporting information (other than missing files) should be addressed to the authors.

Supplementary
