# Supplementary material for: Microwave‐Assisted Selective Hydrogenation of Furfural to Furfuryl Alcohol Employing a Green and Noble Metal‐Free Copper Catalyst
Source: ChemSusChem. 2016 Dec 16;9(24):3387–92. doi: 10.1002/cssc.201601398 (PMC5396339; doi:10.1002/cssc.201601398)
Supplement: Supplementary file 1 — Supplementary [file CSSC-9-3387-s001.pdf]

CHEMISTRY & SUSTAINABILITY
CHEMSUSCHEM
ENERGY & MATERIALS

Supporting Information

Microwave-Assisted Selective Hydrogenation of Furfural to Furfuryl Alcohol Employing a Green and Noble Metal-Free Copper Catalyst

Pedro N. Romano,[a, b] João M. A. R. de Almeida,[a, b] Yuri Carvalho,[a, b] Peter Priecel,[b] Eduardo Falabella Sousa-Aguiar,*[a] and Jose A. Lopez-Sanchez*[b]

cssc_201601398_sm_miscellaneous_information.pdf

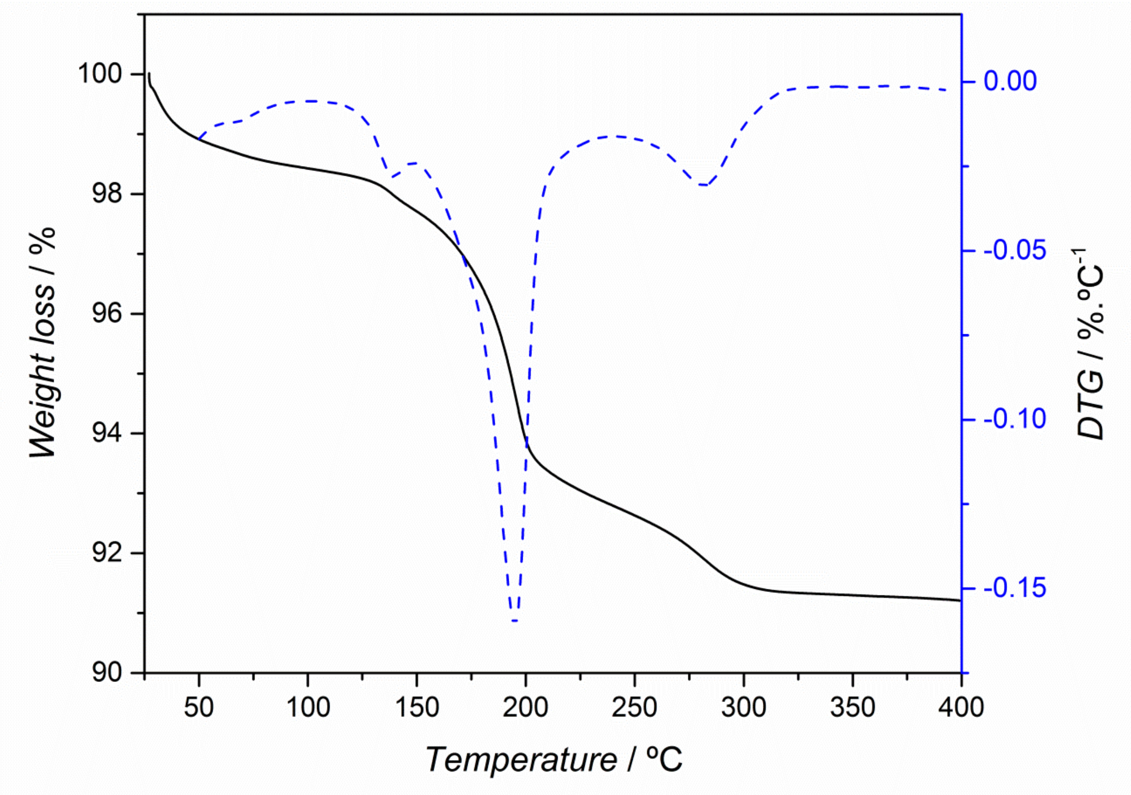

Figure S.1. TGA and DTG curves of the impregnated Cu/TiO_2 catalyst. Experimental conditions: 25 mL/min air flow, 5 ºC/min ramp rate to 400 ºC.

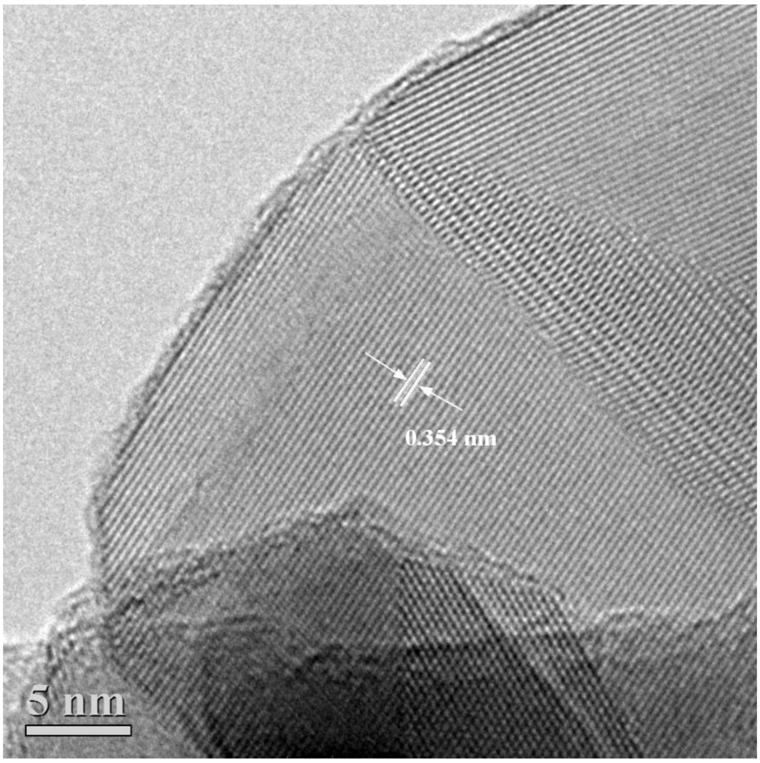

Figure S.2. TEM image of Cu/TiO_2 with the d-spacing of (101) lattice plane of anatase phase highlighted.

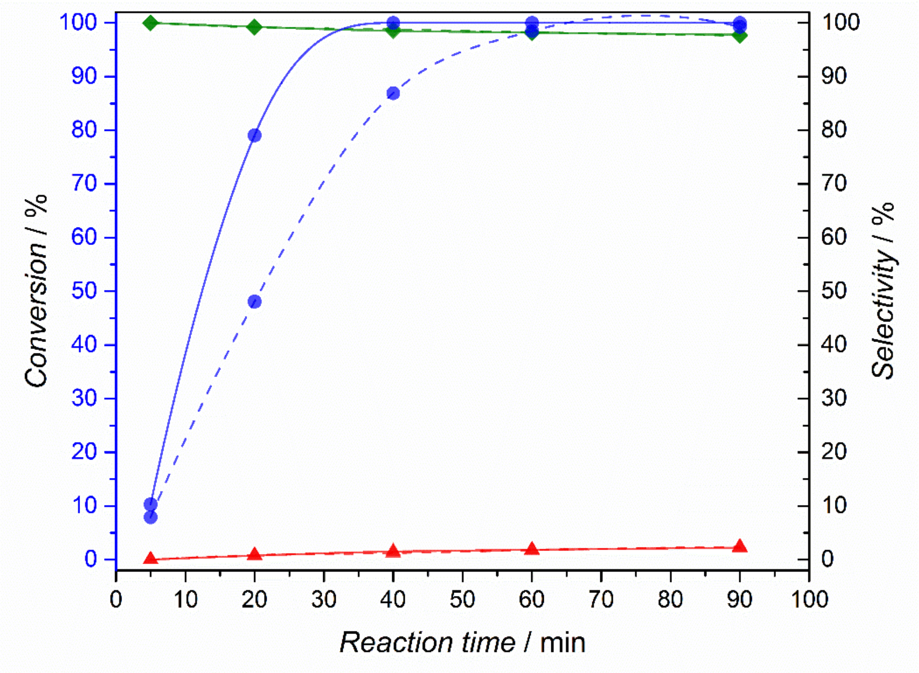

Figure S.3. Comparison between furfural hydrogenation over Cu/TiO_2 under conventional heating (dashed lines) and microwave irradiation (solid lines) at 150 ºC. Legend: conversion (blue line, circle marker), furfuryl alcohol selectivity (green line, diamond marker) and 2-methylfuran selectivity (red line, triangle marker). Parr reactor conditions: 150 ºC; 10 bar of H_2; 60 mg of 10% Cu/TiO_2; 30 mL of a 40 mM FAL solution in CPME. MW reactor conditions: 150 ºC; 10 bar of H_2; 10 mg of 10% Cu/TiO_2; 5 mL of a 40 mM FAL solution in CPME.

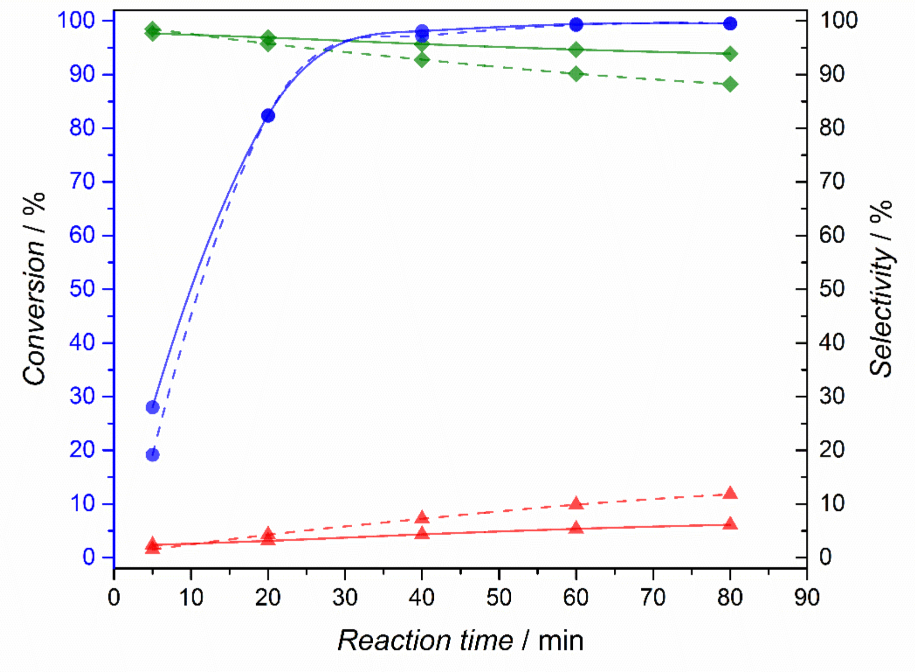

Figure S.4. Comparison between furfural hydrogenation over Cu/TiO_2 under conventional heating (dashed lines) and microwave irradiation (solid lines) at 175 ºC. Legend: conversion (blue line, circle marker), furfuryl alcohol selectivity (green line, diamond marker) and 2-methylfuran selectivity (red line, triangle marker). Parr reactor conditions: 175 ºC; 10 bar of H_2; 60 mg of 10% Cu/TiO_2; 30 mL of a 40 mM FAL solution in CPME. MW reactor conditions: 175 ºC; 10 bar of H_2; 10 mg of 10% Cu/TiO_2; 5 mL of a 40 mM FAL solution in CPME.
